# Evaluation of the Intel RealSense T265 for tracking natural human head motion

**DOI:** 10.1038/s41598-021-91861-5

**Published:** 2021-06-14

**Authors:** Peter Hausamann, Christian B. Sinnott, Martin Daumer, Paul R. MacNeilage

**Affiliations:** 1 Department of Electrical and Computer Engineering, Technical University of Munich, Munich, 80333 Germany; 2 Department of Psychology, University of Nevada, Reno, 89557 USA; 3 Sylvia Lawry Centre for Multiple Sclerosis Research e.V., Munich, 81677 Germany

**Keywords:** Data acquisition, Human behaviour, Motor control

## Abstract

Accurate and robust tracking of natural human head motion in natural environments is important for a number of applications including virtual and augmented reality, clinical diagnostics, as well as basic scientific research. IMU provide a versatile solution for recording inertial data including linear acceleration and angular velocity, but reconstructing head position is difficult or impossible. This problem can be solved by incorporating visual data using a technique known as visual-inertial simultaneous localization and mapping (VI-SLAM). A recently released commercial solution, the Intel RealSense T265, uses a proprietary VI-SLAM algorithm to estimate linear and angular position and velocity, but the performance of this device for tracking of natural human head motion in natural environments has not yet been comprehensively evaluated against gold-standard methods. In this study, we used a wide range of metrics to evaluate the performance of the T265 with different walking speeds in different environments, both indoor and outdoor, against two gold-standard methods, an optical tracking system and a so-called perambulator. Overall, we find that performance of the T265 relative to these gold-standard methods is most accurate for slow to normal walking speeds in small- to medium-sized environments. The suitability of this device for future scientific studies depends on the application; data presented here can be useful in making that determination.

## Introduction

Tracking of human head motion is important across several domains. It is important for investigating basic scientific questions about reflexive control of posture, as well as reflexive stabilization of both head and eye movement^1^. It is also important in applied areas. For example, virtual and augmented reality (VR and AR) rely on tracking of human head motion to render the appropriate visual scene motion in head-mounted displays. And in a clinical setting, one can compare measures of head movement between normal and patient populations to assist in diagnosis and treatment of sensory, motor, and neurological disorders^2^.

Historically, observation of how the head moves in space has been constrained to laboratory settings^3^. In early research, accurate, precise head tracking demanded that the participant wear bulky equipment to track the head mechanically or via magnetic search coil^4–6^. Advances in technology allowed robust head tracking to be conducted with optical tracking systems on humans and other mammals^1,7^. This has been referred to as outside-in head tracking because stationary cameras “outside” the participant are used to track the moving head^8^. This method was more versatile, but robust performance was still confined to the laboratory. More recently, microelectromechanical system (MEMS)-based inertial measurement units (IMUs) have become accessible and affordable enough for widespread use, which in turn has allowed measurement of head movements outside the laboratory^2,9–11^.

MEMS IMUs typically consist of a tri-axial accelerometer and gyroscope, and sometimes a magnetometer, all built into a single small device. These allow estimating linear acceleration, angular velocity, and direction and strength of the local magnetic field, respectively. These estimates may be further processed to estimate orientation relative to gravity, linear velocity and position. Through each of these steps, error is introduced, particularly when integrating and double integrating to estimate linear velocity and position. Estimating orientation is less error-prone because accelerometer, gyroscopes and magnetometers all incorporate information about their orientation with respect to a local reference frame and model-based approaches such as the extended Kalman filter can fuse measurements from all three sensors^12^.

One possibility to address these problems is to incorporate visual data. VI-SLAM is a method developed primarily for use in autonomous robots^13^. The method generally assumes that the IMU and camera(s) are rigidly attached to one another and relies on tracking of visual features of the stationary environment to augment the estimate of linear and angular position derived from IMU data. In the context of virtual and augmented reality, this type of tracking is referred to as inside-out (rather than outside-in) because the sensors mounted on the moving observer are used to track the stationary environment. Optimal algorithms for (VI-SLAM) are an area of active research. However, a commercially available VI-SLAM c recently released and represents a promising tool for versatile tracking of natural human head motion outside the lab. If the T265 device is going to be adopted as a standard tool, its performance must be evaluated. This is especially necessary because only a rough description of the T265’s tracking method is provided^14^; no details about the proprietary closed-source VI-SLAM implementation are available.

Previous studies have compared estimates of position and orientation from the T265 against an optical tracking system (OTS). Alapetite et al.^15^ mounted the device on a wheeled robot and investigated the influence of movement speed as well as the quantity of visual features and moving objects in the environment on the tracking quality. Their results show that tracking performance decreases with higher motion speeds and lower feature density. Ouerghi et al.^16^ evaluated the tracking performance of a hand-held T265 in an industrial environment and measured positional errors below 2% of the overall length of motion trajectories. Agarwal et al.^17^ evaluated the device for indoor navigation of an unmanned aerial system (UAS) and report heading errors of around 3 degrees. Bayer and Faigl^18^ proposed an approach combining the T265 with the RealSense D435 depth camera as a navigation system for a hexapod walking robot and report positional errors of around 10 cm in a laboraty environment. One major gap in the current literature is the evaluation of the T265 for tracking head motion. The device is lightweight (33 g) and affordable ($$\sim $$$ 200), and it is advertised as solution for head tracking for AR and VR. However, to our knowledge, and to date, there are no publicly available studies evaluating its performance in this context.

## Methods

### Evaluation in optical tracking space

**Figure 1:**
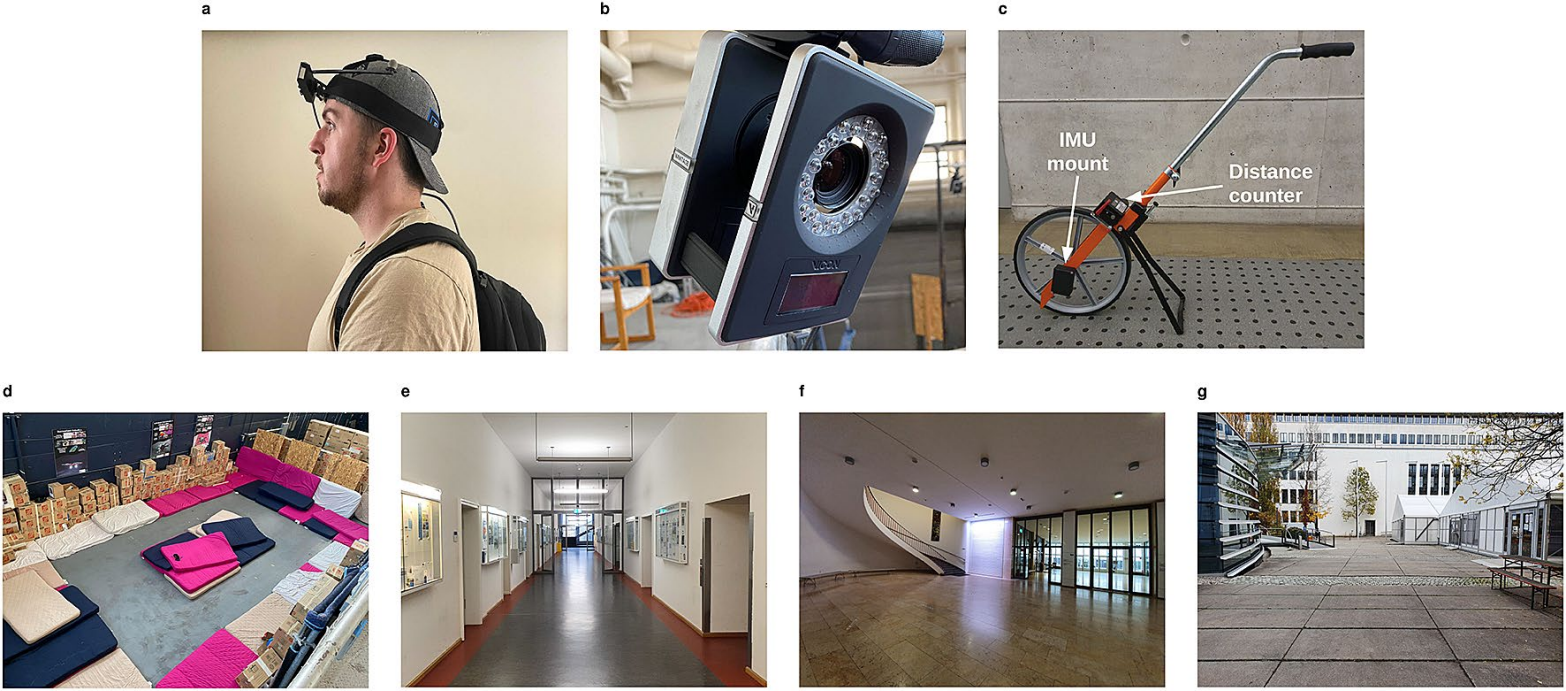
Equipment used for the two studies and snapshots of the four environments where data was recorded (all photographs taken by the authors). (**a**) Subject wearing head mount with T265 and tracking marker. (**b**) One of the Vicon Vantage cameras used in the optical tracking space. (**c**) Perambulator with distance counter and IMU mount. (**d**) Optical tracking space. (**e**) Hallway environment. (**f**) Lobby environment. (**g**) Courtyard environment.

A convenience sample of nine subjects (five female, four male; aged 20–46 years, mean age of 27.8 years) with no known history of vestibular or gait disorders were recruited. All procedures were approved by the Institutional Review Board of the University of Nevada Reno and carried out in accordance with relevant guidelines and regulations. In this study, the pose estimated by the T265 was compared with a gold-standard pose estimate generated by an OTS. A marker was attached to the T265 so that it could be tracked by the OTS. The device and marker were worn by participants on their heads using an elastic headband designed for mounting cameras on the head or helmet during sports activities (Fig. 1a, informed consent to publish the image in an online open-access publication was obtained from the participant).

Participants first performed a synchronization motion by nodding and shaking their head slowly five times each. This data was used to temporally align the T265 and the OTS recordings. Subjects then completed ten laps around the tracking space (Fig. 1d) at three self-chosen speeds: “at a leisurely walking pace”, “at a brisk walking pace”, and “at a jogging pace”. The first five laps for each pace were in a clockwise direction and the last five laps in a counterclockwise direction.

### Evaluation with perambulator

Eight different subjects (three female, five male; aged 26–31 years, mean age of 28 years) with no history of vestibular or gait disorders were recruited for the second part of the study that investigated the speed estimated by the T265 in real-world environments at the main campus of Technical University of Munich (TUM). Here, a so-called perambulator was used as the gold standard measurement device. The perambulator is a surveyor’s wheel (see Fig. 1c)—a device generally used for measuring distances, e.g., in civil engineering—that was modified such that it was also capable of measuring speed^19^. Such a perambulator device has been used in previous studies^20^ in order to measure real-world walking speed of participants. The device is lightweight and can be pushed in an ergonomic manner and thus did not considerably inhibit the normal walking and jogging movements of the participants.

Subjects performed the same set of tasks as in the first study (walk, slow walk, jog) in three different environments while wearing the T265 on the head and pushing the perambulator. The first environment was a hallway ($47 \times 4 \times 5$ m, about 80 m circuit length, Fig. 1e), the second a large lobby ($37 \times 12{-}30 \times 6$ m, about 80 m circuit length, Fig. 1f) and the third a large courtyard ($60 \times 60$ m, surrounded by 5–6 story buildings, about 160 m circuit length, Fig. 1g). Participants were instructed to move along a pre-defined path in each environment. An experimenter took note of the distance measured by the perambulator in each task and environment. All subjects signed an informed consent form compliant with the European General Data Protection Regulation. The study protocol was approved by the institutional review board of the Sylvia Lawry Center for Multiple Sclerosis Research and procedures were carried out in accordance with relevant guidelines and regulations.

### Hardware

The Intel Realsense T265 tracking camera has a diverse suite of sensors which all feed into a VI-SLAM pipeline, which fuses them into a 6 DOF estimation of position and velocity of the camera relative to the environment at 200 Hz. The sensors consist of two global shutter fisheye world cameras (173° diagonal field of view (FOV); $848 \times 800$ pixel resolution; 30 Hz sampling rate), a 3 DOF gyroscope ($\pm 2000  \,\frac{\circ }{s}$ range; 200 Hz sampling rate), and a 3 DOF accelerometer (± 4 g range; 62.5 Hz sampling rate). The 6 DOF estimation of camera position and velocity is computed in real-time onboard the T265 on a dedicated chipset. In both studies, data from the device was recorded via USB with a laptop carried by the participant in a slim backpack.

In the first study, 12 Vicon Vantage 8 cameras were used to perform optical tracking of a rigid body attached to the T265 worn on the head by participants. The Vantage 8 (Fig. 1b) is a purpose-built optical tracking camera produced by Vicon Motion Systems Ltd, UK, capable of recording at 260 Hz with an 8 megapixel resolution. By decreasing the resolution of the camera, the sampling rate can increase to a maximum of 2000 Hz. Each camera has an FOV of 61.7° horizontal by 47° vertical. These cameras created an optical tracking volume measuring $15 \times 8.5 \times 5$ meters, and yielded a 6 DOF pose estimate at 50 Hz.

The perambulator (Fig. 1c) is a modified surveyor’s wheel (Nestle 12006001, Gottlieb Nestle GmbH, Germany) featuring a centimeter-precision distance counter. A housing for an IMU (actibelt RCT3, Trium Analysis Online GmbH, Germany) containing a tri-axial accelerometer ($\pm\, 8$ g range; 100 Hz sampling rate) and gyroscope ($\pm 2000 \; \frac{\circ }{s}$ range; 100 Hz sampling rate) was attached to the axle of the wheel. The gyroscope recorded the instantaneous angular velocity of the wheel which directly corresponds to the speed of the device when being pushed across a surface.

The T265 was worn on the head via a custom-designed 3D-printed mount that holds the T265 securely through two M2.5 screws that thread into the back of the T265. The mount then fastens to an AmazonBasics camera head-strap system available on Amazon (ASIN B00R4YCKIK). In turn, this strap was worn on the head of the participant either directly or over a baseball cap (see Fig. 1a).

### Software

Data from the T265 was recorded with custom software written in Python making use of the pyrealsense2 library developed by Intel (https://github.com/IntelRealSense/librealsense, version 2.36.0). The software recorded the accelerometer and gyroscope streams as well the VI-SLAM position and velocity estimates to disk in a binary format. Information sampled by the OTS cameras was first sent to Vicon Blade software, where a rigid body was fit to the infrared optical marker data. The positional data of this rigid body was then published through the (ROS) middleware via a custom wrapper. Data recorded on the IMU inside the perambulator was read out and processed by a custom software suite written in Julia developed by Trium Analysis Online GmbH.

### Velocity and speed estimation

Linear and angular velocity (${\text {v}}$ and ${ {\omega }}$) were estimated from position (${\text {p}}$) and orientation (${\text {q}}$, in quaternions) provided by the OTS as ${\text {v}} = \dot{{\text {p}}}$ and ${ {\omega }} = {\text{Im}}(2 {\text {q}}^*\dot{{\text {q}}})$. Linear velocity recorded by the T265 as well as the estimate from the OTS (both measured in their respective world frames) were converted to speed by calculating the norm of the earth-horizontal components as $v = \Vert {\text {v}}_{xy} \Vert = \sqrt{v_x^2 + v_y^2}$.

The angular velocity $\omega _P$ measured by the perambulator was filtered with a fourth-order Butterworth low-pass filter with a cutoff frequency of 10 Hz. Then it was transformed to linear speed by multiplying the angular velocity component in the direction of the axle with the circumference of the wheel ($C=1\text {m}$) as $v = C \cdot \omega _P$. Finally, samples with $v<0.01$ m/s and segments shorter than 3 s were removed from the estimate.

### Time synchronization

The timestamps of the data collected from the OTS were corrected by computing the cross-correlation function of the angular velocity ${ {\omega }}$ with that measured by the T265 ($\hat{{{\omega }}}$) during the calibration segment (see “Evaluation in optical tracking space” section). The temporal lag $\Delta t$ of the maximum of this function was determined with $K = {\text{argmax}}_k \sum _i^n \Vert \hat{{ {\omega }}}_i\Vert \cdot \Vert { {\omega }}_{i+k}\Vert$ and $\Delta t = \hat{t}_K - t_K$ and the timestamps of the T265’s measurements were shifted by this amount.

In the second study, the perambulator’s IMU was tapped against the T265 at the beginning of each recording. This created visually distinguishable peaks in the accelerometer measurements of both devices. The timestamps of these peaks were used to manually correct the time offset.

For both studies, data recorded from the T265 was interpolated to match the timestamps of the respective gold standard (perambulator or OTS) after temporal alignment. A simple linear interpolation was used for position as well as linear and angular velocity. Orientation, expressed in quaternions, was interpolated using the spherical quadrangle method^21^.

### Reference frame transformations

The OTS provides position and orientation of the tracked rigid body with respect to its world frame *W* (denoted ${}^{W}\!{\text {p}}$ and ${}^{W}\!{\text {q}}$). The T265 provides its own position and orientation as well as linear and angular velocity with respect to a different world frame $\hat{W}$ (denoted ${}^{\hat{W}}\!\hat{{\text {p}}}$, ${}^{\hat{W}}\!\hat{{\text {q}}}$, ${}^{\hat{W}}\!\hat{{\text {v}}}$ and ${}^{\hat{W}}\!\hat{{ {\omega }}}$).

The transformation between the world frames *W* and $\hat{W}$ was estimated using a basic point set registration (PSR) method^16^ which was used to transform position and orientation of the T265 from its own to the OTS world frame. A rotation-only PSR method was used to estimate the transformation from the body frames *B* and $\hat{B}$ to calibrated frames *C* and $\hat{C}$ that are independent of the orientation of the head mount on the subject’s head. For the T265, we achieved this calibration by calculating the rotation that simultaneously centers heading direction (i.e., the direction of instantaneous linear velocity) along the longitudinal axis (*x*) and gravity direction along the vertical axis (*z*). The OTS was calibrated by aligning linear velocity and gravity direction to those measured by the T265 in its calibrated frame with the same rotation-only PSR method. Details on these estimations and transformations can be found in the Supplementary Material.

**Figure 2:**
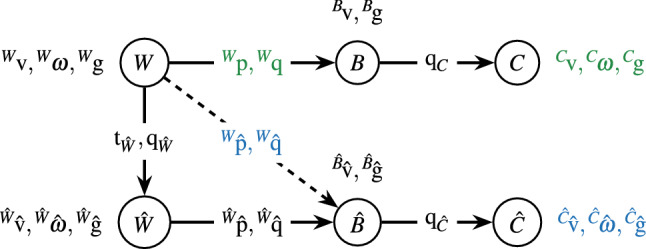
Reference frame tree with corresponding measurements and transformations. The top row shows the relationship between world (*W*), body (*B*) and calibrated (*C*) frame of the OTS as well as the measurements used in the following analysis highlighted in green. The second row shows the relationship between world ($\hat{W}$), body ($\hat{B}$) and calibrated ($\hat{C}$) frame of the T265 as well as the measurements used in the following analysis highlighted in blue. The dashed arrow denotes the estimate of the T265’s position and orientation with respect to the OTS world frame.

The above estimations (T265 world frame and calibrated frames) were performed for each subject and each task during the first 30 s of each task. The transformations obtained from these estimations were then applied to all measurements recorded during the task. This ensured that enough data was available for a robust estimation while at the same time reducing the possibility of drifts in position and orientation influencing the result. The complete reference frame tree with all transformations between frames is shown in Fig. 2. Unless specified otherwise, positions and orientations reported below are represented in the world frame *W* (e.g., $\hat{{\text {p}}}$ as a shorthand for ${}^{W}\!\hat{{\text {p}}}$) while velocities and accelerations are represented in the respective calibrated body frames (e.g., $\hat{{\text {v}}}$ as a shorthand for ${}^{\hat{C}}\!\hat{{\text {v}}}$).

### Performance metrics

The primary aim of this study was to compare the position and velocity estimates provided by the T265 with those provided by the OTS and perambulator. This comparison was conducted using a number of metrics to quantify specific aspects of performance. An overview of these performance metrics is shown in Table 1.

**Table 1:** Description of performance metrics. Metrics denoted with a star ($*$) were computed for both studies, all other metrics only for the optical tracking space study. For metrics denoted with a dagger ($\dagger$) we excluded samples where the gold-standard motion speed was below 0.1 m/s.

Performance metric	Unit	Description	Formula
TLE$^{*}$	%	Relative difference between trajectory length estimated by T265 and gold standard length	$\frac{\hat{L} - L}{L}$
ATE	m	RMS of distances between position estimated by T265 and OTS across the full trajectory^22^	$\sqrt{\frac{1}{n} \sum _i^n \left\| \hat{{\text {p}}}_i - {\text {p}}_i \right\| ^2}$
RTE	m	RMS of relative distance between position estimated by T265 and OTS over a window of *k* samples^22^	$$\sqrt{\frac{1}{n} \sum _i^n \begin{aligned}\textstyle \Vert&\hat{{\text {p}}}_{i+k} - \hat{{\text {p}}}_i \\&\;\;-{\text{rot}}(\hat{{\text {q}}}_{\gamma ,i}\cdot {\text {q}}_{\gamma ,i}^{-1}, {\text {p}}_{i+k} - {\text {p}}_i) \Vert ^2 \end{aligned}}$$
TDr	%	Distance between final position estimates of a trajectory relative to trajectory length^22^	$\frac{\left\| \hat{{\text {p}}}_n - {\text {p}}_n \right\| }{L}$
GDE	°	Mean angle between representation of gravity vectors in the respective calibrated frames	$\frac{1}{n} \sum _i^n \arccos {\frac{\langle \hat{{\text {g}}}_i, {\text {g}}_i \rangle }{\Vert \hat{{\text {g}}}_i\Vert \cdot \Vert {\text {g}}_i\Vert }}$
${\text{GDE}}-\alpha$, ${\text{GDE}}-\beta$	°	Roll and pitch angle difference	$\hat{\alpha } - \alpha$, $\hat{\beta } - \beta$
AYE	°	RMS of yaw angle difference across full trajectory^22^	$\sqrt{\frac{1}{n} \sum _i^n \Delta \gamma _i^2}$
RYE	°	RMS of yaw angle difference over a window of *k* samples^22^	$\sqrt{\frac{1}{n} \sum _i^n \left( \Delta \gamma _{i+k} - \Delta \gamma _i \right) ^2}$
YDr	°/h	Final yaw angle difference relative to trajectory duration *T*^22^	$\frac{\Delta \gamma _n}{T}$
SpE$^{*,\dagger }$	%	Mean relative difference between earth-horizontal speed measured by the T265 and gold standard speed	$\frac{1}{n} \sum _i^n \frac{\hat{v}_i - v_i}{v_i}$
HDE$^{\dagger }$	°	Mean angle between linear velocity vectors estimated by T265 and OTS	$\frac{1}{n} \sum _i^n \arccos {\frac{\langle \hat{{\text {v}}}_i, {\text {v}}_i \rangle }{\Vert \hat{{\text {v}}}_i\Vert \cdot \Vert {\text {v}}_i\Vert }}$
${\text{HDE}}-\theta$, ${\text{HDE}}-\phi$ $^{\dagger }$	°	Azimuth and elevation angle diff.	$\hat{\theta }_v - \theta _v$, $\hat{\phi }_v - \phi _v$
LVME$^{\dagger }$	m/s	Mean difference between magnitudes of linear velocity estimated by T265 and OTS	$\frac{1}{n} \sum _i^n \Vert \hat{{\text {v}}}_i\Vert - \Vert {\text {v}}_i\Vert$
AVDE$^{\dagger }$	°	Mean angle between angular velocity vectors estimated by T265 and OTS	$\frac{1}{n} \sum _i^n \arccos {\frac{\langle \hat{{ {\omega }}}_i, { {\omega }}_i \rangle }{\Vert \hat{{ {\omega }}}_i\Vert \cdot \Vert { {\omega }}_i\Vert }}$
${\text{AVDE}}-\theta$, ${\text{AVDE}}-\phi$ $^{\dagger }$	°	Azimuth and elevation angle diff.	$\hat{\theta }_\omega - \theta _\omega$, $\hat{\phi }_\omega - \phi _\omega$
AVME$^{\dagger }$	°/s	Mean difference between magnitudes of angular velocity estimated by T265 and OTS	$\frac{1}{n} \sum _i^n \Vert \hat{{ {\omega }}}_i\Vert - \Vert { {\omega }}_i\Vert$

The trajectory length *L* was computed from position data provided by T265 and OTS as the sum of earth-horizontal displacements, i.e., the norm of the difference in *x* and *y* direction between two consecutive samples: $L = \sum _i^n \left\| {\text {p}}_{xy,i} - {\text {p}}_{xy,i-1} \right\|$. Since the position estimate by the T265 is occasionally subject to re-localization jumps that would result in an over-estimation of the trajectory length, samples where the instantaneous speed was above 5 m/s were considered artifacts and excluded from this computation. Trajectory length measured by the perambulator was directly provided by the distance counter of the device.

For the calculation of the relative translation error (RTE), yaw drift at the beginning of the window was removed by rotating the position estimate of the OTS with ${\text{rot}}(\hat{{\text {q}}}_{\gamma ,i}\cdot {\text {q}}_{\gamma ,i}^{-1}, {\text {p}}_{i+k} - {\text {p}}_i)$. Here, ${\text{rot}}({\text {q}}, {\text {v}}) = {\text {q}} {\text {v}} {\text {q}}^{-1}$ denotes the rotation of a vector ${\text {v}}$ by the quaternion ${\text {q}}$ and ${\text {q}}_\gamma = [ \sqrt{1 - q_z^2}, 0, 0, q_z ]^\top$ denotes the quaternion representing the yaw component of ${\text {q}}$. Since the OTS data was recorded at a sampling rate of 50 Hz, we used a window length of $k=50$ to obtain windows of approximately 1 second. The same window length was used for the calculation of the (RYE).

The roll ($\alpha$) and pitch ($\beta$) angle with respect to gravity were computed as $\alpha = \arctan {{g_y}/{g_z}}$ and $\beta = -\arcsin {{g_x}/{\Vert {\text {g}}\Vert }}$. Difference in yaw angle was computed as the geodesic distance between the yaw components of the orientations from T265 and OTS as $\Delta \gamma = \arccos {( 2 < \hat{{\text {q}}}_\gamma , {\text {q}}_\gamma >^2 - 1 )}$^23^. We excluded outliers in the first and 99th percentile of $\Delta \gamma$ from further analysis.

Heading is the instantaneous direction of linear velocity in head coordinates. Heading elevation ($\phi _v$) and azimuth ($\theta _v$) angle were computed as $\phi _v = \arcsin {{v_z}/{\Vert {\text {v}}\Vert }}$ and $\theta _v = -\arctan {{v_y}/{v_x}}$. We also computed angular velocity elevation ($\phi _\omega = \arcsin {{\omega _z}/{\Vert { {\omega }}\Vert }}$) and azimuth angle ($\theta _\omega = -\arctan {{\omega _y}/{\omega _x}}$). This corresponds to the direction of the instantaneous axis of rotation.

### Statistical analysis

Distributions of motion speeds *v* where estimated using a kernel density estimate (KDE) with $\hat{f}_h(v) = {1}/{nh}\sum _i^n K({v-v_i}/{h})$^24^. We used a Gaussian kernel $K(x) = {1}/{\sqrt{2\pi }}\,e^{{-x^2}/{2}}$ and a bandwidth of $h=0.2$, manually chosen for visual representation (see Fig. 8).

We used a one-way repeated measures analysis of variance (ANOVA) to determine whether the means of error metrics were significantly different across tasks. We reported the *F*-statistic as well as *p*-values and considered effects significant if $p<0.05$, in which case we performed a two-sided paired t-test between all pairs of tasks as a post-hoc test. For this test, we reported *p*-values corrected with the Bonferroni method and divided by 2 (to obtain a one-sided result dependent on the *t*-statistic, see below), considering differences significant when $p<0.05$. Additionally, we deemed metrics to be larger in the first task of the pair if the *t*-statistic was positive and smaller if the *t*-statistic was negative. In the perambulator study, we performed a two-way repeated measures ANOVA in the same manner to determine the influence of task and environment on the error metrics, combined with a post-hoc paired *t*-test across tasks and environments in the case of significant effects. We did not compare metrics between the two studies since the difference in gold standard as well as environment and test subjects were confounding factors we could not control for. It should also be noted that this is an exploratory study, therefore the significance of these inferential statistics might be somewhat limited.

All statistical analyses were performed in Python 3.6. We used the statsmodels library (version 0.12.1) for the ANOVA and the scipy library (version 1.5.3) for the KDE and *t*-tests. Error metrics were plotted for different tasks and environments using boxplots. Boxes were plotted from the first to the third quartile with the band indicating the median. Whiskers indicated the range from the lowest sample within 1.5 times the interquartile range (IQR) of the lower quartile to the highest sample within 1.5 times the IQR of the upper quartile.

## Results

**Figure 3:**
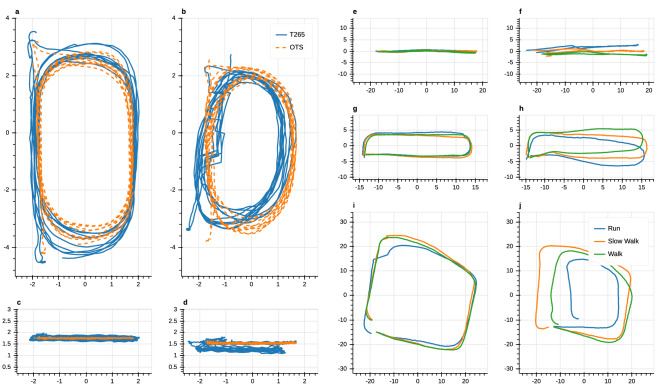
Example trajectories recorded in all environments. All axes indicate position in m; axis labels were omitted to avoid cluttering. (**a–d**) Top and side view of trajectories recorded in the optical tracking space comparing position estimated by T265 (blue) and OTS (orange, dashed). (**a,c**) Successful tracking during walking. (**b,d**) Unsuccessful tracking exhibiting re-localizations and drift in both vertical direction and yaw during running. (**e–j**) Top view of example trajectories recorded in the real-world environments comparing position estimated by T265 across different tasks (blue: running, orange: slow walking, green: walking). (**e**) Successful tracking in hallway environment. (**f**) Unsuccessful tracking in hallway environment exhibiting yaw drift and re-localization. (**g**) Successful tracking in lobby environment. (**h**) Unsuccessful tracking in lobby environment exhibiting yaw drift. (**i**) Successful tracking in courtyard environment. (**j**) Unsuccessful tracking in courtyard environment exhibiting yaw drift and task-dependent under-estimation of displacement.

A comparison of trajectories recorded from T265 and OTS in the optical tracking space is shown in Fig. 3a–d. The figure demonstrates a case of successful tracking during walking (panels a and c) and a failure case during running (panels c,d). While there is an overestimation of displacement in the first case, the second case exhibits significant drift both in yaw angle (b) and vertical direction (d). Jumps in the T265 trajectory in Fig. 3b are due to re-localization of the device by means of loop closure, i.e., a correction of the current pose estimate based on re-observation of previously observed landmarks.

Figure 3e–j compares the earth-horizontal components of trajectories reported by the T265 in the perambulator study. The left column shows successful tracking cases in the hallway (panel e), lobby (panel g) and courtyard (panel h) environment while the right column (panels f, h and i) shows failure cases. The unsuccessful cases are characterized by yaw drift and, in the courtyard example (j), a task-dependent under-estimation of displacement.

**Figure 4:**
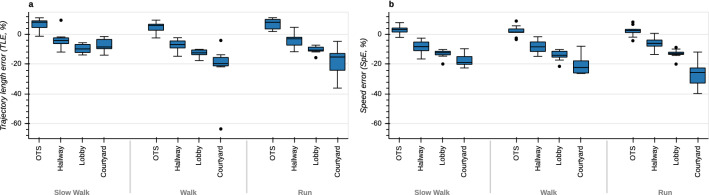
Boxplots of trajectory length and speed errors between T265 and gold standard across different environments and tasks. (**a**) Trajectory length errors. (**b**) Speed errors.

TLE are shown in Fig. 4a. Median trajectory length errors (TLEs) in the OTS study are positive, indicating a task-dependent over-estimation of trajectory length ($F(2,16)=10.84, p=0.001$) that is higher during slow walking ($p=0.013$) and running ($p=0.005$) compared to walking. TLE in the perambulator study are dependent on environment ($F(2,14)=10.08, p=0.002$) and task ($F(2,14)=7.75, p=0.005$). Median values are negative and under-estimation is significantly smaller in the hallway environment when compared with the lobby ($p=0.013$) and courtyard environments ($p=0.015$). Additionally, we observe more under-estimation during running ($p=0.012$) and walking ($p=0.007$) compared to slow walking.

**Figure 5:**
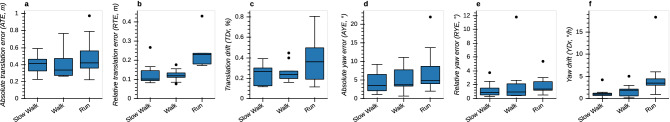
Boxplots of translation and yaw errors between T265 and OTS across different tasks. (**a**) Absolute translation error. (**b**) Relative translation error. (**c**) Translation drift. (**d**) Absolute yaw error. (**e**) Relative yaw error. (**f**) Yaw drift.

Positional data was also used to quantify absolute and relative translation error (ATEs, RTEs) and yaw error (AYEs, RYEs) as well as drift in translation (TDrs) and yaw (YDrs, Fig. 5). Median absolute translation errors (ATEs) are around 0.4 m and values do not depend on task ($F(2,16)=1.77, p=0.202$, Fig. 5a). RTE are dependent on task ($F(2,16)=26.57, p<0.001$) and significantly higher in the running task than in the walking ($p=0.002$) and slow walking task ($p<0.001$, Fig. 5b). Median translation drifts (TDrs) are between 0.2 and 0.4 m/h and values are not task-dependent ($F(2,16)=2.35, p=0.128$, Fig. 5c). Median Median absolute yaw errors (AYEs) are between 3 and 5° and are not dependent on task ($F(2,16)=3.09, p=0.073$, Fig. 5d). Median RYEs increased from 0.9 to 1.4°from slow walking to running, although the overall effect is not statistically significant ($F(2,16)=0.46, p=0.637$, Fig. 5e). YDr depend on task ($F(2,16)=6.98, p=0.007$) and are higher during running than slow walking ($p=0.034$, medians between 1 and 3.5°/h, Fig. 5f).

**Figure 6:**
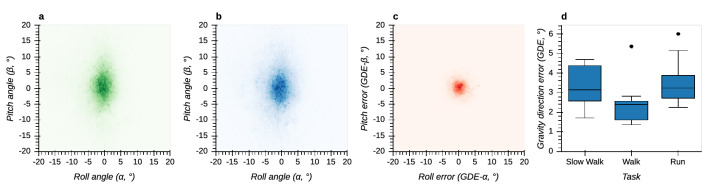
Gravity direction measured by T265 and OTS and corresponding errors. Positive pitch angles correspond to forward pitch, positive roll angles to rightward roll. (**a**) Bi-variate histogram of pitch and roll angles measured by the OTS. (**b**) Bi-variate histogram of pitch and roll angles measured by the T265. (**c**) Bi-variate histogram of pitch and roll errors between T265 and OTS. (**d**) Boxplot of gravity direction errors between T265 and OTS across different tasks.

Orientation relative to gravity as tracked by both T265 and OTS shows an elongated distribution around the pitch axis (Fig. 6a and b). The gravity direction errors (GDEs) are relatively small and similar for both pitch and roll axes (Fig. 6c). Median values are between 1.6 and 3° and depend on task ($F(2,16)=8.49, p=0.003$). Specifically, they are smaller in the walking task in comparison with the slow walking ($p=0.026$) and running task ($p=0.014$, Fig. 6d).

**Figure 7:**
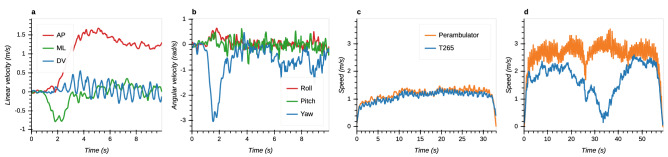
Example time series of velocities measured by T265. (**a**) Linear velocity in AP, ML and DV directions in optical tracking space during walking. (**b**) Angular velocity around roll, pitch and yaw axes in optical tracking space during walking. (**c**) Comparison between speed measured by T265 and perambulator during walking in hallway environment showing successful tracking, see Fig. 3e. (**d**) Comparison between speed measured by T265 and perambulator during running in courtyard environment showing unsuccessful tracking with considerable under-estimation of speed, see Fig. 3j.

Linear and angular velocity measures were also compared. Example traces from both studies are shown in Fig. 7. 3-DOF linear and angular velocities of a participant measured by the T265 while walking in the optical tracking space are displayed in Fig. 7a,b. Panels c,d compare earth-horizontal movement speed calculated from the T265 data with the gold standard perambulator measurements. Fig. 7c demonstrates a case of successful tracking in the hallway environment during walking, corresponding to the trajectory shown in Fig. 3e. In contrast, Fig. 7d illustrates a failure case characterized by a severe under-estimation of motion speed during running in the courtyard environment that corresponds to the trajectory in Fig. 3j.

**Figure 8:**
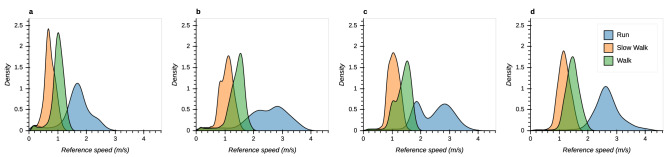
KDE of gold standard speed across different environments and tasks (blue: running, orange: slow walking, green: walking). (**a**) Optical tracking space. (**b**) Hallway environment. (**c**) Lobby environment. (**d**) Courtyard environment.

Movement speeds are strongly dependent on task, both in the optical tracking space ($F(2,16)=219.98, p<0.001$) and in the real-world environments ($F(2,14)=129.62, p<0.001$, all post-hoc tests yielded $p<0.001$, Fig. 8). Median speed during slow walking ranges from 0.7 m/s in the optical tracking space (Fig. 8a) to 1.2 m/s in the courtyard environment (Fig. 8d). Similarly, median values of walking and running speed range from 1.0 to 1.5 m/s and from 1.7 to 2.6 m/s, respectively. Running speed, especially in the hallway and lobby environments, shows a bi-modal distribution (Fig. 8b,c). However, there is no significant effect of environment on movement speed in the perambulator study ($F(2,14)=3.72, p=0.051$).

Median speed errors (SpEs) are close to 0% in the optical tracking space and do not depend on task ($F(2,16)=2.10, p=0.155$, Fig. 4b). In the perambulator study, SpEs are negative and decrease with increasing size of the environment ($F(2,14)=27.22, p<0.001$), with significantly lower values in the courtyard environment when compared to the hallway ($p=0.001$) and lobby environments ($p=0.009$). Additionally, we observe significantly lower values in the lobby in comparison with the hallway ($p=0.024$). This indicates a tendency of the T265 to under-estimate speed in larger, more complex environments and is consistent with the results for TLEs. The error is also significantly dependent on task ($F(2,14)=4.52, p=0.031$), where we observe more under-estimation during running compared to slow walking ($p=0.028$).

**Figure 9:**
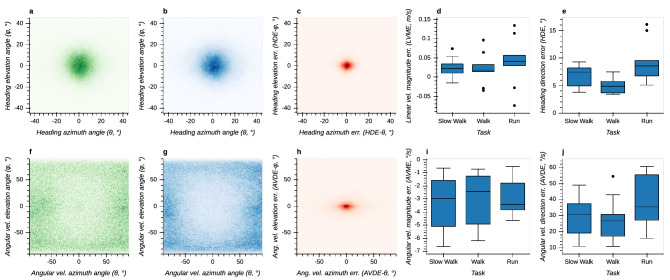
Linear and angular velocity measured by T265 and OTS and corresponding errors. (**a**) Bi-variate histogram of heading azimuth and elevation angles measured by the OTS. Positive elevation angles correspond to upward heading, positive azimuth angles to rightward heading. (**b**) Bi-variate histogram of heading azimuth and elevation angles measured by the T265. (**c**) Bi-variate histogram of heading azimuth and elevation errors between T265 and OTS. (**d**) Boxplot of linear velocity magnitude errors between T265 and OTS across different tasks. (**e**) Boxplot of heading direction errors between T265 and OTS across different tasks. (**f**) Bi-variate histogram of angular velocity azimuth and elevation angles measured by the OTS. Positive elevation corresponds to a leftward rotation around the yaw axis, positive azimuth to an upward rotation around the pitch axis. (**g**) Bi-variate histogram of azimuth and elevation angles measured by the T265. (**h**) Bi-variate histogram of azimuth and elevation errors between T265 and OTS. (**i**) Boxplot of angular velocity magnitude errors between T265 and OTS across different tasks. (**j**) Boxplot of angular velocity direction errors between T265 and OTS across different tasks.

Heading directions are centered around 0° elevation and azimuth angles (Fig. 9a,b). Error in heading direction (HDEs) shows a centered distribution, with similar extents in elevation and azimuth (Fig. 9c) and is dependent on task ($F(2,16)=8.32, p=0.003$, Fig. 9e). Errors are lowest in the walking task with a median value of about 4°and significantly smaller compared to the slow walking ($p=0.005$) and running task ($p=0.012$). Errors in the magnitude of the linear velocity vector (LVMEs) are centered close to 0°/s and do not depend on task ($F(2,16)=2.39, p=0.123$, Fig. 9d).

Angular velocity directions (i.e., the axes of rotation) are distributed towards $\pm\, 90$° elevation and azimuth angles (Fig. 9f,g). This indicates that the instantaneous axis of head rotation is more frequently aligned with the pitch and yaw axis than with the roll axis (which corresponds to zero azimuth and elevation). Error in angular velocity direction (AVDEs) shows a centered distribution that is elongated in the azimuth direction (Fig. 9h). Overall, errors are dependent on task ($F(2,16)=5.55, p=0.015$), but the post-hoc analysis revealed no significant differences (Fig. 9j). Errors in the magnitude of angular velocity (AVDEs) are centered around − 3°/s and are not task-dependent ($F(2,16)=1.41, p=0.273$, Fig. 9i).

## Discussion

Measurement of natural human head motion in natural environments is important for a range of applications including VR/AR technology, clinical diagnostics, as well as basic scientific investigation of sensorimotor function. If VI-SLAM devices such as the T265 are going to be used for these applications, their accuracy must be evaluated. That is the primary aim of this study. Which measures of human head position and motion are most important varies greatly across applications. Therefore, we have evaluated accuracy using a wide range of metrics. Performance was evaluated relative to two gold-standard methods, the OTS because it can estimate all 6-DOF of head position and the perambulator because it can be used in any environment, including outdoors. We measured performance for a range of locomotor speeds because speed impacts both IMU data and visual data and may also impact how the VI-SLAM algorithm estimates linear and angular position. We also measured performance for a range of environments because environmental features are known to impact the reliability of visual data used for VI-SLAM.

Regarding the effect of environment, both trajectory length and movement speed are underestimated by the T265 relative to the perambulator and this error increases with the size of the environment (Fig. 4). This effect is likely to reflect underestimation of the physical scale or size of the visual scene and thus underestimation of the distance and speed of human movement. Large environment size can pose difficulties for many VI-SLAM algorithms^13,25^. As the environment increases in size, landmarks used by the T265’s VI-SLAM algorithm may increasingly get further from the cameras. In turn, the resultant landmark movement used to estimate camera motion may be underestimated due to the decreased stereo disparity of these landmarks sensed by the T265’s cameras. Future studies using the T265 to measure ground speed and distance of linear head motion in diverse environments should be aware of this potential source of inaccuracy; other devices, such as the perambulator may be preferable in this context.

Some metrics are also affected by locomotor speed. Several of these show a monotonic increase in error with increasing locomotor speed. With the perambulator as gold-standard, underestimation of trajectory length is greater during running and walking than during slow-walking (Fig. 4). With the OTS as gold-standard, relative translation error is greater during running compared to walking and slow-walking; Yaw drift error is also higher during running than slow-walking (Fig. 5). These monotonic effects of speed are likely due to noise on VI-SLAM signals that increases with locomotor speed. For example, increased speed may lead to motion blur in the visual data which may hinder landmark localization. Regarding IMU data, noise may also increase with increased power at higher frequencies. Another factor is the relatively low camera frame rate of 30 Hz. With higher movement speed, tracked landmarks can move considerably within the camera image between consecutive frames, which in turn might degrade the tracking performance. Finally, the VI-SLAM algorithm itself may be optimized to operate best during slower, smoother motions.

In contrast, other metrics show a non-monotonic effect of locomotor speed. Specifically, gravity direction error (Fig. 6) and heading direction error (Fig. 9) are both smallest during normal walking and greater for slow walking and running. The similar pattern of results for these two metrics may be because they both depend on how sum total linear acceleration, sensed by the accelerometer, is partitioned into gravitational and inertial components. The fact that performance is best for normal walking may be because the T265 algorithm has been specifically tuned to perform best during normal walking to facilitate its use in VR/AR applications. Unfortunately, it is not possible to verify these speculations because the T265 VI-SLAM algorithm is proprietary. Nevertheless, this pattern of results suggests that the T265 is a suitable choice for applications that require estimation of heading and gravity direction during normal walking.

These results are in line with those reported by Alapetite et al.^15^ that suggest that motion speed and density of visual features in the environment have the greatest effect on the T265’s performance while the presence of moving objects has less impact. We did not explicitly investigate the effect of feature richness as our focus was on real-world environments of varying size, although it could be argued that visual features are sparser in larger environments. In a future study, it would be interesting to evaluate the tracking performance in a feature-poor real-world setting such as a meadow. Our results regarding accuracies in position and heading angle also seem to confirm previous reports by Agarwal et al.^17^, Bayer and Faigl^18^, and Ouerghi et al.^16^. In the former, the authors note that the tracking performance of the T265 increases throughout multiple runs across the same environment which suggests that the device stores the features of a number of recently observed visual landmarks.

One limitation of the current study is the inability to calculate most performance metrics in more realistic, outdoor environments. To accomplish this, it would be necessary to use a gold-standard method that can measure linear and angular position in a large, naturalistic outdoor space. Unfortunately we did not have access to a large-scale, outdoor OTS or other method that would be suitable for this purpose. As a consequence, we were not able to evaluate how many of our metrics are affected by tracking in larger, outdoor environments. For example, we might expect yaw drift to be greater in larger environments due to the inability of the VI-SLAM algorithm to achieve loop closure (see, e.g., Fig. 3b and d). This was most likely not a problem in the small optical tracking space. Yaw drift and possibly other metrics measured in this space may not reflect performance in larger spaces.

Another limitation is the inability to evaluate what amount of measured error should be attributed to inaccuracy of the gold-standard. In particular, the T265 uses a gyroscope to measure angular velocity and this inertial measure of angular velocity may be more reliable than the angular velocity estimate provided by the OTS. Also, the measures derived from the perambulator are known to be less accurate when the walking trajectory is curved. We tried to mitigate this by instructing subjects to make curves as large as possible and, in the hallway environment, lifting up the perambulator and turning around in place at the end of the hallway. It is also possible that the additional cognitive load of pushing the perambulator while walking or jogging caused participants to bias or change their own locomotion in some unforeseen way. Despite these possible limitations, the gold-standard methods used here are generally much more accurate than the T265 meaning that our metrics generally reflect performance of the T265 more than performance of the gold-standard method.

Moving forward, it would be important to further investigate in more detail which environmental features are most likely to cause tracking failures by the T265 or by VI-SLAM systems generally. Relevant environmental features include variation in light level of the environment, presence of independently moving objects, and environmental size or scale. These can all impact the ability of the VI-SLAM algorithm to identify, track, and use visual landmark features. Conventional SLAM systems often mitigate these issues through incorporation of additional sensors such as global positioning system (GPS), light detection and ranging (LIDAR), and wheel odometry^25^. While many early SLAM algorithms are highly redundant, using many landmarks, modern VI-SLAM methods limit the amount of landmarks used in order to increase computational efficiency^26^, requiring greater emphasis on landmark selection and removal of erroneous or unusable landmarks. Recent efforts to combine machine learning techniques with VI-SLAM largely serve these needs, and decrease error driven by dynamic features^27^. The T265 does not include any sensors other than those previously described, and at time of writing it is unknown how the T265 performs feature selection.

An alternative to VI-SLAM that avoids environmental dependence is positional tracking based on purely non-visual data. The simplest form of non-visual tracking is based on inertial data only. Such tracking usually yields more noisy positional estimates, although certain biomechanical constraints such as the assumption of zero foot velocity during the stance phase can be used to correct drifts and integration errors^28^. Ongoing research is aimed at improving inertial tracking for the specific application of tracking human head position^22^. It would be possible to compare the tracking performance of the T265’s VI-SLAM algorithm to an estimate computed only from its raw IMU data. However, this is subject to algorithm selection and parameter tuning which is why we deemed this comparison to be out of scope for the current study.

In principle, it would also be possible to improve the estimation yielded by the T265 by performing VI-SLAM post-hoc. While one of the larger selling points of the T265 is real-time VI-SLAM, it is not necessary for scientific investigation of human head motion. Measurements taken from the sensors of the T265 could be saved and subsequently passed through customized VI-SLAM algorithms that are optimized for the specific application of tracking natural human head motion in natural environments.

In summary, the T265 appears to be best suited for tracking human head position during normal walking in small- to medium-sized environments with limited dynamic features. Increases (and sometimes decreases) in locomotor speed tend to increase observed error, as does use of the system in larger and more dynamic outdoor environments. The acceptability of the default, factory-set performance of the T265 depends on the application. In future, customized VI-SLAM algorithms may be applied to data collected by the T265 or other devices post-hoc such that estimation is optimized for tracking natural human head movement in natural environments. Additional evaluation studies in larger, dynamic, and outdoor environments would be very helpful for tuning of such custom VI-SLAM algorithms.

## Supplementary Information

Supplementary Information.

## Data Availability

The data recorded in both studies is available at https://gin.g-node.org/phausamann/t265-evaluation.

## References

[1] Pozzo T, Berthoz A, Lefort L (1989). Head kinematic during various motor tasks in humans. Prog. Brain Res..

[2] Hausamann P, Daumer M, MacNeilage PR, Glasauer S (2019). Ecological momentary assessment of head motion: Toward normative data of head stabilization. Front. Hum. Neurosci..

[3] MacNeilage P, Fritsch B, Straka H (2020). Characterization of natural head movements in animals and humans. The Senses: A Comprehensive Reference.

[4] Bartz AE (1966). Eye and head movements in peripheral vision: Nature of compensatory eye movements. Science.

[5] Barnes GR (1979). Vestibulo-ocular function during co-ordinated head and eye movements to acquire visual targets. J. Physiol..

[6] Crane BT, Demer JL (1997). Human gaze stabilization during natural activities: Translation, rotation, magnification, and target distance effects. J. Neurophysiol..

[7] Malinzak MD, Kay RF, Hullar TE (2012). Locomotor head movements and semicircular canal morphology in primates. Proc. Natl. Acad. Sci. U.S.A..

[8] Pustka, D. *et al.* Optical outside-in tracking using unmodified mobile phones. In *ISMAR 2012—11th IEEE International Symposium on Mixed and Augmented Reality 2012, Science and Technology Papers* 81–89 (2012). 10.1109/ISMAR.2012.6402542.

[9] MacDougall HG (2005). Marching to the beat of the same drummer: The spontaneous tempo of human locomotion. J. Appl. Physiol..

[10] Mayerhoffer A, MacNeilage P (2011). Natural Statistics of Vestibular Stimulation During Human Locomotion.

[11] Carriot J, Jamali M, Cullen KE, Chacron MJ (2017). Envelope statistics of self-motion signals experienced by human subjects during everyday activities: Implications for vestibular processing. PLoS ONE.

[12] Sabatini AM (2006). Quaternion-based extended Kalman filter for determining orientation by inertial and magnetic sensing. IEEE Trans. Biomed. Eng..

[13] Fuentes-Pacheco J, Ruiz-Ascencio J, Rendón-Mancha JM (2012). Visual simultaneous localization and mapping: A survey. Artif. Intell. Rev..

[14] Grunnet-Jepsen, A. *et al.**Introduction to Intel RealSense Visual SLAM and the T265 Tracking Camera* (2019).

[15] Alapetite A, Wang Z, Hansen JP, Zajaçzkowski M, Patalan M (2020). Comparison of three off-the-shelf visual odometry systems. Robotics.

[16] Ouerghi, S., Ragot, N., Boutteau, R. & Savatier, X. Comparative study of a commercial tracking camera and ORB-SLAM2 for person localization. In *VISIGRAPP 2020—Proceedings of the 15th International Joint Conference on Computer Vision, Imaging and Computer Graphics Theory and Applications*, Vol. 4, 357–364 (2020). 10.5220/0008980703570364.

[17] Agarwal, A., Crouse, J. R. & Johnson, E. N. Evaluation of a commercially available autonomous visual inertial odometry solution for indoor navigation. In *2020 International Conference on Unmanned Aircraft Systems, ICUAS 2020* 372–381 (2020). 10.1109/ICUAS48674.2020.9213962.

[18] Bayer, J. & Faigl, J. On autonomous spatial exploration with small hexapod walking robot using tracking camera Intel RealSense T265. In *2019 European Conference on Mobile Robots (ECMR)* 1–6 (IEEE, 2019). 10.1109/ECMR.2019.8870968.

[19] Aigner G, Grimm B, Lederer C, Daumer M (2019). Method to collect ground truth data for walking speed in real-world environments: Description and validation. PeerJ Prepr..

[20] Schimpl M, Lederer C, Daumer M (2011). Development and validation of a new method to measure walking speed in free-living environments using the actibelt® platform. PLoS ONE.

[21] Shoemake, K. Quaternion calculus and fast animation, computer animation: 3-D motion specification and control. In *SIGGRAPH 1987 Tutorial* 101–121 (Siggraph, 1987).

[22] Liu W (2020). TLIO: Tight learned inertial odometry. IEEE Robot. Autom. Lett..

[23] Huynh DQ (2009). Metrics for 3D rotations: Comparison and analysis. J. Math. Imaging Vis..

[24] Silverman, B. W. *Density Estimation: For Statistics and Data Analysis* (2018).

[25] Scaramuzza D, Fraundorfer F (2011). Tutorial: Visual odometry. IEEE Robot. Autom. Mag..

[26] Bailey T, Durrant-Whyte H (2006). Simultaneous localization and mapping (SLAM): Part II. IEEE Robot. Autom. Mag..

[27] Bahraini MS, Rad AB, Bozorg M (2019). SLAM in dynamic environments: A deep learning approach for moving object tracking using ML-RANSAC algorithm. Sensors (Switzerland).

[28] Foxlin E (2005). Pedestrian tracking with shoe-mounted inertial sensors. IEEE Comput. Graph. Appl..

