# Supplementary material for: Evaluation of the Intel RealSense T265 for tracking natural human head motion
Source: Sci Rep. 2021 Jun 14;11:12486. doi: 10.1038/s41598-021-91861-5 (PMC8203655; doi:10.1038/s41598-021-91861-5)
Supplement: Supplementary file 1 — Supplementary Information. [file 41598_2021_91861_MOESM1_ESM.pdf]

Evaluation of the Intel RealSense T265 for Tracking Natural Human Head Motion

Supplementary material

Peter Hausamann[1,*], Christian B. Sinnott[2], Martin Daumer[1,3], and Paul R. MacNeilage[2]

[1]Technical University of Munich, Department of Electrical and Computer Engineering, Munich, 80333, Germany
[2]University of Nevada, Department of Psychology, Reno, 89557, United States of America
[3]Sylvia Lawry Centre for Multiple Sclerosis Research e.V., Munich, 81677, Germany
[*]peter.hausamann@tum.de

Details of reference frame transformations

The transformation between the world frame W of the optical tracking system (OTS) and that of the T265 ($\hat{W}$) was estimated using a basic point set registration (PSR) method. The method estimates a rotation matrix R and a translation vector t for two sets $\mathscr{X} = \{\mathrm{x}_i \mid i \in 1..n\}$ and $\mathscr{Y} = \{\mathrm{y}_i \mid i \in 1..n\}$ of corresponding points that minimizes $\sum_i^n \|\mathrm{y}_i - (\mathrm{R}\mathrm{x}_i + \mathrm{t})\|_2$.

$$\mathrm{H} = \sum_i^n (\mathrm{x}_i - \bar{\mathrm{x}})(\mathrm{y}_i - \bar{\mathrm{y}})^\top \tag{1a}$$

$$\mathrm{U}, \mathrm{S}, \mathrm{V} = \mathrm{SVD}(\mathrm{H}) \tag{1b}$$

$$\mathrm{R} = \mathrm{V}\mathrm{U}^\top \tag{1c}$$

$$\mathrm{t} = -\mathrm{R}\bar{\mathrm{y}} + \bar{\mathrm{x}} \tag{1d}$$

where $\bar{\mathrm{x}} = \frac{1}{n}\sum_i^n \mathrm{x}_i$ and $\bar{\mathrm{y}} = \frac{1}{n}\sum_i^n \mathrm{y}_i$ denote the centroids of $\mathscr{X}$ and $\mathscr{Y}$, respectively. A modified version this algorithm with $\mathrm{H} = \sum_i^n \mathrm{x}_i \mathrm{y}_i$ can be used to estimate a transform that consists only of a rotation and minimizes $\sum_i^n \|\mathrm{y}_i - \mathrm{R}\mathrm{x}_i\|_2$.

With $\mathscr{X} = \{{}^{\hat{W}}\hat{\mathrm{p}}_i \mid i \in 1..n\}$ and $\mathscr{Y} = \{{}^{W}\mathrm{p}_i \mid i \in 1..n\}$ this yields $\mathrm{R}_{\hat{W}}$ and $\mathrm{t}_{\hat{W}}$ which can be used to transform position and orientation of the T265 from its own to the OTS world frame:

$${}^{W}\hat{\mathrm{p}} = \mathrm{rot}\left(\mathrm{q}_{\hat{W}}, {}^{\hat{W}}\hat{\mathrm{p}}\right) + \mathrm{t}_{\hat{W}} \tag{2a}$$

$${}^{W}\hat{\mathrm{q}} = \mathrm{rot}\left(\mathrm{q}_{\hat{W}}, {}^{\hat{W}}\hat{\mathrm{q}}\right) \tag{2b}$$

Here, $\mathrm{rot}(\mathrm{q}, \mathrm{v}) = \mathrm{q}\mathrm{v}\mathrm{q}^{-1}$ denotes the rotation of a vector v by the quaternion q and $\mathrm{q}_{\hat{W}}$ the equivalent quaternion representation of $\mathrm{R}_{\hat{W}}$.

The modified PSR method was used to estimate the transformation from the body frames B and $\hat{B}$ to calibrated frames C and $\hat{C}$ that are independent of the orientation of the head mount on the subject's head. In the case of the T265, this calibration was achieved by calculating the rotation that centers heading direction along the longitudinal x-axis and gravity direction along the vertical z-axis. For this, we first computed the representation of the gravity vector ${}^{\hat{W}}\mathrm{g}$ - a unit length vector pointing upwards in the vertical direction in world coordinates - as well as the linear velocity vector in the body frame $\hat{B}$:

$${}^{\hat{B}}\mathrm{g} = \mathrm{rot}\left({}^{\hat{W}}\hat{\mathrm{q}}, {}^{\hat{W}}\mathrm{g}\right) \tag{3a}$$

$${}^{\hat{B}}\hat{\mathrm{v}} = \mathrm{rot}\left({}^{\hat{W}}\hat{\mathrm{q}}, {}^{\hat{W}}\hat{\mathrm{v}}\right) \tag{3b}$$

The modified PSR algorithm on the sets

$$\mathscr{X} = \left\{{}^{\hat{B}}\hat{\mathrm{v}}_i \;\middle|\; i \in 1..n\right\} \cup \left\{{}^{\hat{B}}\mathrm{g}_i \;\middle|\; i \in 1..n\right\} \tag{4a}$$

$$\mathscr{Y} = \left\{[\|\hat{\mathrm{v}}_i\|, 0, 0]^\top \;\middle|\; i \in 1..n\right\} \cup \left\{[0, 0, \|\mathrm{g}_i\|]^\top \;\middle|\; i \in 1..n\right\} \tag{4b}$$

yields the rotation matrix $\mathrm{R}_{\hat{C}}$ and its equivalent quaternion representation $\mathrm{q}_{\hat{C}}$ which was used to transform the linear and angular velocity as well as the gravity vector measured by the T265 into $\hat{C}$:

$$^{\hat{C}}\hat{\mathrm{v}} = \mathrm{rot}\left({}^{\hat{W}}\hat{\mathrm{q}} \cdot \mathrm{q}_{\hat{C}}, {}^{\hat{W}}\hat{\mathrm{v}}\right) \tag{5a}$$

$$^{\hat{C}}\hat{\boldsymbol{\omega}} = \mathrm{rot}\left({}^{\hat{W}}\hat{\mathrm{q}} \cdot \mathrm{q}_{\hat{C}}, {}^{\hat{W}}\hat{\boldsymbol{\omega}}\right) \tag{5b}$$

$$^{\hat{C}}\hat{\mathrm{g}} = \mathrm{rot}\left({}^{\hat{W}}\hat{\mathrm{q}} \cdot \mathrm{q}_{\hat{C}}, {}^{\hat{W}}\mathrm{g}\right) \tag{5c}$$

The OTS was calibrated by aligning linear velocity and gravity direction to those measured by the T265 in its calibrated frame by running the modified PSR on the sets

$$\mathscr{X} = \left\{ {}^{\hat{B}}\hat{\mathrm{v}}_i \,\middle|\, i \in 1..n \right\} \cup \left\{ {}^{\hat{B}}\mathrm{g}_i \,\middle|\, i \in 1..n \right\} \tag{6a}$$

$$\mathscr{Y} = \left\{ {}^{B}\mathrm{v}_i \,\middle|\, i \in 1..n \right\} \cup \left\{ {}^{B}\mathrm{g}_i \,\middle|\, i \in 1..n \right\} \tag{6b}$$

to obtain the rotation matrix R_C and its equivalent quaternion representation q_C. With this, we transformed the linear and angular velocity of the marker tracked by the OTS as well as the gravity vector into C:

$$^{C}\mathrm{v} = \mathrm{rot}\left({}^{W}\mathrm{q} \cdot \mathrm{q}_C, {}^{W}\mathrm{v}\right) \tag{7a}$$

$$^{C}\boldsymbol{\omega} = \mathrm{rot}\left({}^{W}\mathrm{q} \cdot \mathrm{q}_C, {}^{W}\boldsymbol{\omega}\right) \tag{7b}$$

$$^{C}\mathrm{g} = \mathrm{rot}\left({}^{W}\mathrm{q} \cdot \mathrm{q}_C, {}^{W}\mathrm{g}\right) \tag{7c}$$

All estimations and transformations were performed in Python 3.6 using the `rigid-body-motion` library (version 0.3.0, https://github.com/phausamann/rigid-body-motion).
